# Effect of Twist-Drill Craniostomy With Hollow Screws for Evacuation of Chronic Subdural Hematoma: A Meta-Analysis

**DOI:** 10.3389/fneur.2021.811873

**Published:** 2022-01-28

**Authors:** Zeng Wei, Haixiao Jiang, Ying Wang, Cunzu Wang

**Affiliations:** ^1^Medical College of Yangzhou University, Yangzhou University, Yangzhou, China; ^2^Northern Jiangsu People's Hospital (NJPH), Yangzhou, China

**Keywords:** SEPs, the hollow screw, YL-1 needle, chronic subdural hematoma, meta-analysis

## Abstract

**Objective:**

This study systematically reviews the clinical efficacy and safety of twist-drill craniostomy with hollow screws in chronic subdural hematoma treatment.

**Methods:**

A computerized search of PubMed, Embase, Web of Science, Cochrane Library, World Health Organization International Trial Registry platform, CBM, CNKI, and Wanfang Database was performed to retrieve randomized controlled trials or case-control trials using twist-drill craniostomy (TDC) with hollow screws for the evacuation of chronic subdural hematoma from the date of databases' inception to July 2021. Two investigators independently screened the studies and extracted data in strict accordance with pre-established inclusion and exclusion criteria. RevMan 5.3 software or STATA was used for meta-analysis after evaluating the methodological quality of the included studies.

**Results:**

A total of 4 randomized controlled trials and 16 case-control trials with a total of 2,536 cases were included. Results of the meta-analysis showed that the surgical success rate and postoperative recurrence rate of TDC with hollow screws were slightly higher compared to the burr hole craniostomy (BHC) group, but showed no statistical significance (RR = 1.03, *P* = 0.05; RR = 1.13, *P* = 0.50). However, subgroup analysis showed that the use of YL-1 needle had a higher success rate and lower recurrence rate (RR = 1.05, *P* = 0.02 < 0.05; RR = 0.584, *P* = 0.002), and TDC with hollow screws had a lower incidence rate of postoperative complications and postoperative acute intracranial hemorrhage compared with BHC, also revealing an overall shorter hospital stay (RR = 0.57, *P* = 0.0002 < 0.05; RR = 0.584, *P* = 0.027 < 0.05; WMD = −3.752, *P* < 0.001). However, the postoperative mortality rate was practically the same between the two groups (OR = 1.01, *P* = 0.95 > 0.05).

**Conclusion:**

Twist-drill craniostomy with hollow screws is not inferior or superior to BHC in efficacy, and this strategy is safer and minimally invasive, which is reflected in a lower incidence of acute intracranial hemorrhage, overall complication rate, and length of hospital stay.

**Systematic Review Registration:**

https://www.crd.york.ac.uk/prospero/, identifier: CRD42021270835.

## Introduction

Chronic subdural hematoma (CSDH) is one of the most common diseases encountered in the neurosurgical department. The incidence of CSDH has been reported to be 1.72–20.60 per 100,000 people, and the elderly population accounts for the large majority. Moreover, due to the aging of the global population, some scholars have observed an increased incidence of CSDH (1–5). The main feature of CSDH is the accumulation of blood and its by-products in the subdural space; therefore, the progression of CSDH is relatively slow (6). The surgical evacuation of hematoma remains the primary treatment method of CSDH. There are three commonly used surgical methods in clinical practice to date, including burr hole craniostomy (BHC), twist-drill craniostomy (TDC), and craniotomy. BHC and TDC are used as primary options, while craniotomy is generally used for acute subdural hematoma or extensive CSDH and is considered as a secondary option for most patients with CSDH (7, 8). There is still a debate on whether BHC or TDC is better. The evidence-based studies by Weigel et al. (9) and Lega et al. (10) suggested that BHC is safer and more effective than TDC. However, with the improvement of TDC, especially TDC with hollow screws connected to a closed drainage system has emerged. TDC with hollow screws, a bedside procedure, utilizes a special puncture needle to drill and penetrate the skull and dura mater directly to the hematoma cavity, and the closed drainage system is reconnected through the twist-drill hole on the skull without the need to insert a catheter into the subdural space. There is increasing evidence that TDC with hollow screws should be the first-line intervention clinically, but most of the published data are from single institutional experiences. There are three kinds of puncture needles currently available: the Subdural Evacuating Port System (SEPS, initially manufactured by Medical Designs LLCA and later by Medtronic Inc.), the hollow screw (initially manufactured by Fehling Instruments and later by Teleflex Medical), and YL-1 needle (manufactured by Beijing WanTeFu Medical Apparatus). Although Hoffman et al. (11) and Chari et al. (12) have done evidence-based research on the TDC with hollow screws, their studies were on SEPS only. Moreover, the study by Chari et al. is relatively old and did not include the findings on TDC that utilized YL-1 needles. Furthermore, both did not include the results of randomized controlled trials. Hence, this study systematically reviews the clinical efficacy and safety of TDC with hollow screws (including SEPS, the hollow screw, and YL-1 needle) in CSDH treatment, thereby providing a reference for clinical diagnosis and treatment.

## Materials and Methods

### Search Strategy

A systematic literature search of PubMed, Embase, Web of Science, Cochrane Library, World Health Organization International Trial Registry platform, CBM, CNKI, and Wanfang Database was performed. Keywords used included “Hematoma, Subdural, Chronic, Craniotomy, YL-1 needle, hollow screws, minimally invasive puncture drainage, Twist-drill craniostomy, subdural evacuating port system, SEPS, randomized controlled trial, and Case-Control Studies.” The search was performed by combining subject headings with free words, and the publication time was not restricted. The search strategy was determined after multiple independent pre-searches by two investigators.

### Study Selection

Inclusion criteria included: (1) study design: the trial was designed as a randomized controlled trial or case-control trial with more than 10 patients (prospective and retrospective). The intervention was a primary procedure to treat CSDH with TDC with hollow screws (i.e., SEPS, the hollow screw, and the YL-1 needle); (2) subjects: any patient with chronic or subacute subdural hematoma (imaging diagnosis: mainly crescentic iso-dense, low-density, or mixed density shadows beneath the inner table of the skull and on the brain surface seen on cranial CT imaging); (3) outcome measures: main outcome measures included a) surgical success rate: many studies had different definitions for “success” and lacked long-term follow-up results. We defined surgical success as follows: the clinical symptoms were almost or complete disappeared with the imageology showed that the hematoma was removed obviously (the volume was lessen over 50%); b) a recurrence rate of postoperative hematoma; and c) postoperative complications. Secondary outcome measures included acute postoperative bleeding, mortality, length of hospital stay, and hospital costs; (4) the languages were restricted to Chinese and English.

Exclusion criteria included: (1) studies with only an abstract and lacking a full text with no response from the authors when contacted; (2) studies with incomplete data or the data that could not be calculated from the original study and that were not described in detail; (3) two studies with similar results from the same institution; (4) subjects who suffered from other diseases besides CSDH, such as cerebral hemorrhage, epidural hematoma, severe systemic disease, severe liver and kidney dysfunction, acute hemorrhage, and brain hernia; (5) subjects with a history of intracranial hemorrhage, infection, tumors, and craniotomy.

### Data Extraction and Quality Appraisal

Two investigators independently screened the studies and completed the data extraction by screening the title, abstract, and full text in strict accordance with the pre-defined inclusion and exclusion criteria. The extracted contents included the first author, publication time, sample size, baseline conditions, intervention, and outcome measures. Two investigators then cross-checked the results, and the studies with conflicting opinions were decided upon after discussion by the research team. The quality of randomized controlled trials was appraised using the Cochrane systematic review criteria, and the quality of non-randomized case-control studies was appraised using the Newcastle–Ottawa scoring system (NOS; including selection, comparability, and exposure or outcome, and the highest quality studies are awarded up to nine stars).

### Statistical Analysis

Meta-analysis was performed using RevMan 5.3 software or STATA provided by the Cochrane Collaboration. Relative risk (RR) was used for data enumeration and standardized mean difference (WMD) was used for data measurement, and 95% confidence interval was also calculated. The heterogeneity of the included studies was tested, and the χ^2^-test was employed to analyze the statistical heterogeneity. If *I*^2^ ≤ 50% (*P* < 0.1), it indicated that the heterogeneity was small, and a fixed-effect model was used for meta-analysis. If *I*^2^ > 50% (*P* > 0.1), it indicated that the heterogeneity was significant, and the sources of heterogeneity were analyzed by L' Abbe plot, radial plot, sensitivity analysis, meta-regression, and clinical analysis. Furthermore, subgroup analysis was performed when necessary. If no clinical heterogeneity was present, a random-effect model was used for meta-analysis. Funnel plots and Egger's test were also used to analyze potential publication bias.

## Results

### Search Results

**Figure 1:**
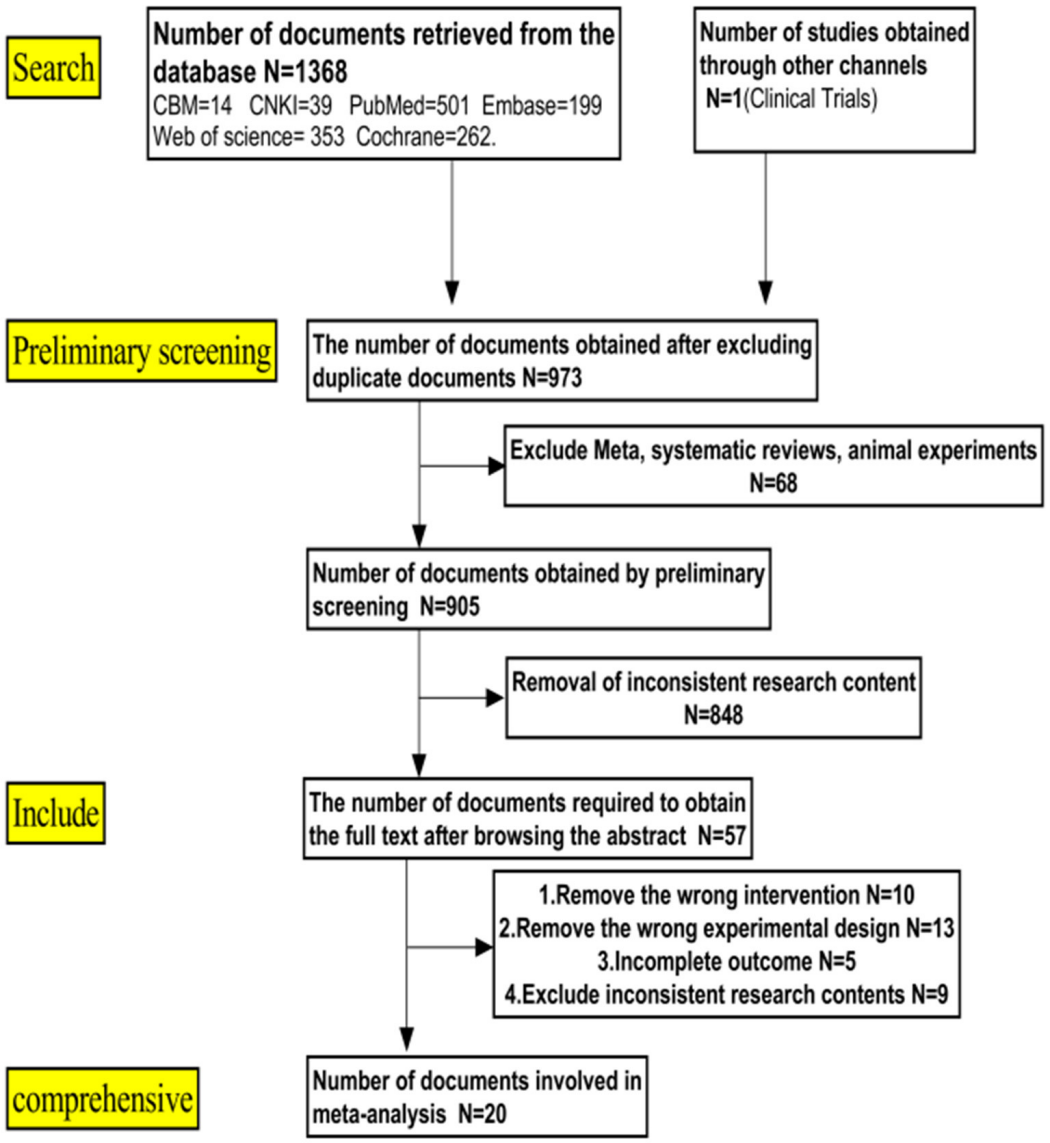
Literature screening process.

A total of 1,369 studies were obtained in the initial search, and 20 studies (13–32) were finally included based on the inclusion and exclusion criteria. These 20 studies included 17 English articles (13–20, 24–32) and 3 Chinese articles (21–23), of which four of them were randomized controlled trials (15, 17, 20, 21) while the remaining 16 were case-control trials. The workflow of literature retrieval and screening is shown in Figure 1.

### Characteristics of the Included Studies

Twenty studies with a total of 2,536 cases were included, including 1,114 cases in the experimental group and 1,422 in the control group. The baseline of patients was comparable. All experimental groups of the included studies were treated with TDC with hollow screws (SEPS, the hollow screw, or YL-1 needle) for CSDH, while the control groups were treated with BHC. The basic characteristics of the included studies are listed in Table 1.

**Table 1:** Basic characteristics of included studies.

**References**	**Number of cases**		**Gender (male/female)**		**Age (years)**		**Intervention**		**Follow-up**		**Hospital costs**	
											**T**	**C**
	**T**	**C**	**T**	**C**	**T**	**C**	**T**	**C**	**T**	**C**	**108391 ± 11124($)**	**166318 ± 21452($)**
Golub et al. (13)	39	68	29/10	48/22	68.7 ± 2.4	66.8 ± 2.2	S	B	3W		NA	
Muzii et al. (15)	22	24	14/8	16/8	78.7	76.3	H	B	2M		NA	
Rughani et al. (16)	21	21	14/7	14/7	73	73.3	S	B	66.8D	45D	NA	
Singh et al. (17)	48	52	43/5	47/5	59.8	61.2	H	B	3M		NA	
Szmuda et al. (18)	5	27	3/2	20/7	69	72.2	H	B	NA		8685.98 ± 1531.42	11759.85 ± 2363.21(¥)
Wang et al. (19)	68	53	57/11	44/9	69.49 ± 12.69	66.67 ± 13.13	Y	B	1M		NA	
Xu et al. (20)	20	20	16/4	17/3	66.20 ± 10.11	66.00 ± 16.74	Y	B	3M		NA	
Teng et al. (21)	30	30	16/14	17/13	62.1 ± 0.47	62.09 ± 0.48	Y	B	1M		NA	
Wang et al. (19)	28	38	NA	68.2 ± 18.5	67.3 ± 12.9	H	B		3M		NA	
Liu et al. (22)	17	44	16/1	41/3	57	60	Y	B	3M		NA	
Zhu et al. (23)	66	69	55/11	60/9	65	61	Y	B	3M		NA	
Flint et al. (24)	371	659	257/117	464/195	75	76	S	B	6M		NA	
Ortiz et al. (25)	25	41	NA	71.5	71.3	S	B		NA		NA	
Xu et al. (26)	116	42	101/15	33/9	65.3 ± 14.2	67.5 ± 14.56	Y	B	3M		NA	
Safain et al. (27)	23	23	15/8	14/9	68	61	H	B	12W	15W	NA	
Smely et al. (28)	33	33	21/12	21/12	69.7	70	H	B	81D	82 D	NA	
Gabarros et al. (29)	105	83	NA	NA	S	B	1y		NA	
Balser et al. (30)	29	44	29/0	44/0	76.6	78.4	H	B	3y		48446 ± 33226($)	67227 ± 6457($)
Wan et al. (31)	31	31	19/12	18/13	72.5	73.6	Y	B	3M		NA	
Fei et al. (32)	17	19	11/6	15/4	85	75	Y	B	6M		NA	

*S, the Subdural Evacuating Port System; B, burr hole craniotomy; Y, YL-1 needle; D, day; W, week; M, month; y, year*.

### Quality Appraisal

**Figure 2:**
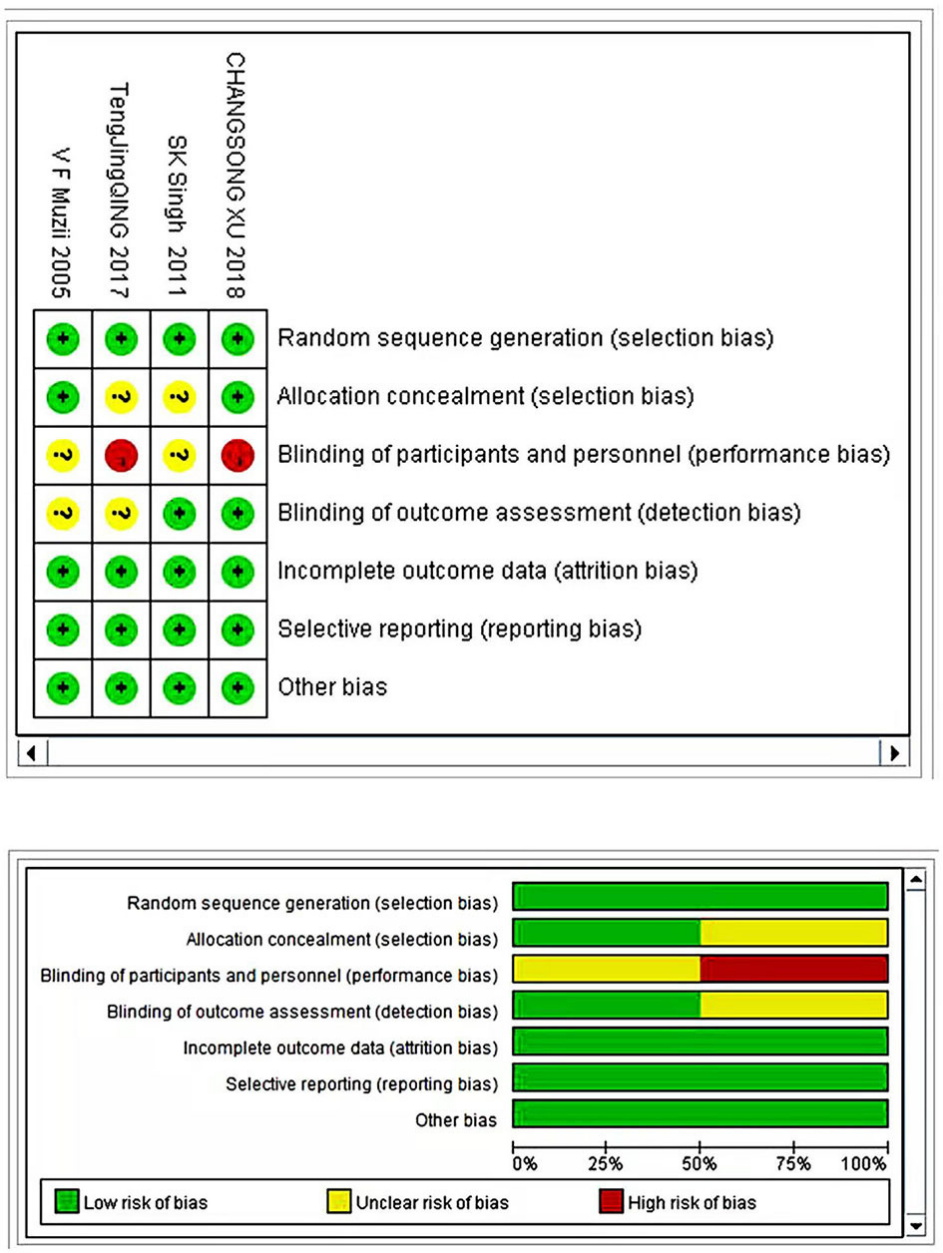
Cochrane assessment result for risk of bias.

Four randomized controlled studies reported random allocation methods with complete results data, no selective report, no conflict of interest, and a balanced baseline. Only two studies reflected the allocation concealment (15, 20), and similarly, only two articles reported the implementation of blinding (17, 20) (Figure 2). All 16 case-control trials were scored using the NOS scoring system. Eight studies were scored as eight stars, three were scored as seven stars, three were scored as six stars, and two articles were scored as five stars (Table 2).

**Table 2:** Quality assessment of NOS.

**References**	**Selection**	**Comparability**	**Outcome**	**Total**
Golub et al. (13)	⋆⋆⋆	⋆⋆	⋆⋆⋆	8
Wang et al. (14)	⋆⋆⋆	⋆⋆	⋆⋆⋆	8
Rughani et al. (16)	⋆⋆⋆	⋆⋆	⋆⋆⋆	8
Szmuda et al. (18)	⋆⋆		⋆⋆⋆	5
Wang et al. (19)	⋆⋆⋆	⋆⋆	⋆⋆⋆	8
Liu et al. (22)	⋆⋆		⋆⋆⋆	5
Zhu et al. (23)	⋆⋆⋆		⋆⋆⋆	6
Flint et al. (24)	⋆⋆⋆	⋆⋆	⋆⋆⋆	8
Ortiz et al. (25)	⋆⋆⋆		⋆⋆⋆	6
Xu et al. (26)	⋆⋆	⋆⋆	⋆⋆⋆	7
Safain et al. (27)	⋆⋆⋆		⋆⋆⋆	6
Smely et al. (28)	⋆⋆⋆	⋆⋆	⋆⋆⋆	8
Gabarros et al. (29)	⋆⋆⋆	⋆⋆	⋆⋆⋆	8
Balser et al. (30)	⋆⋆⋆	⋆	⋆⋆⋆	7
Wan et al. (31)	⋆⋆⋆	⋆⋆	⋆⋆⋆	8
Fei et al. (32)	⋆⋆⋆	⋆	⋆⋆⋆	7

*NOS used the semi-quantitative principle of star system to evaluate the quality of literature, with a full score of 9 “⋆, ⋆⋆, ⋆⋆⋆”*.

### Meta-Analysis Results

**Figure 3:**
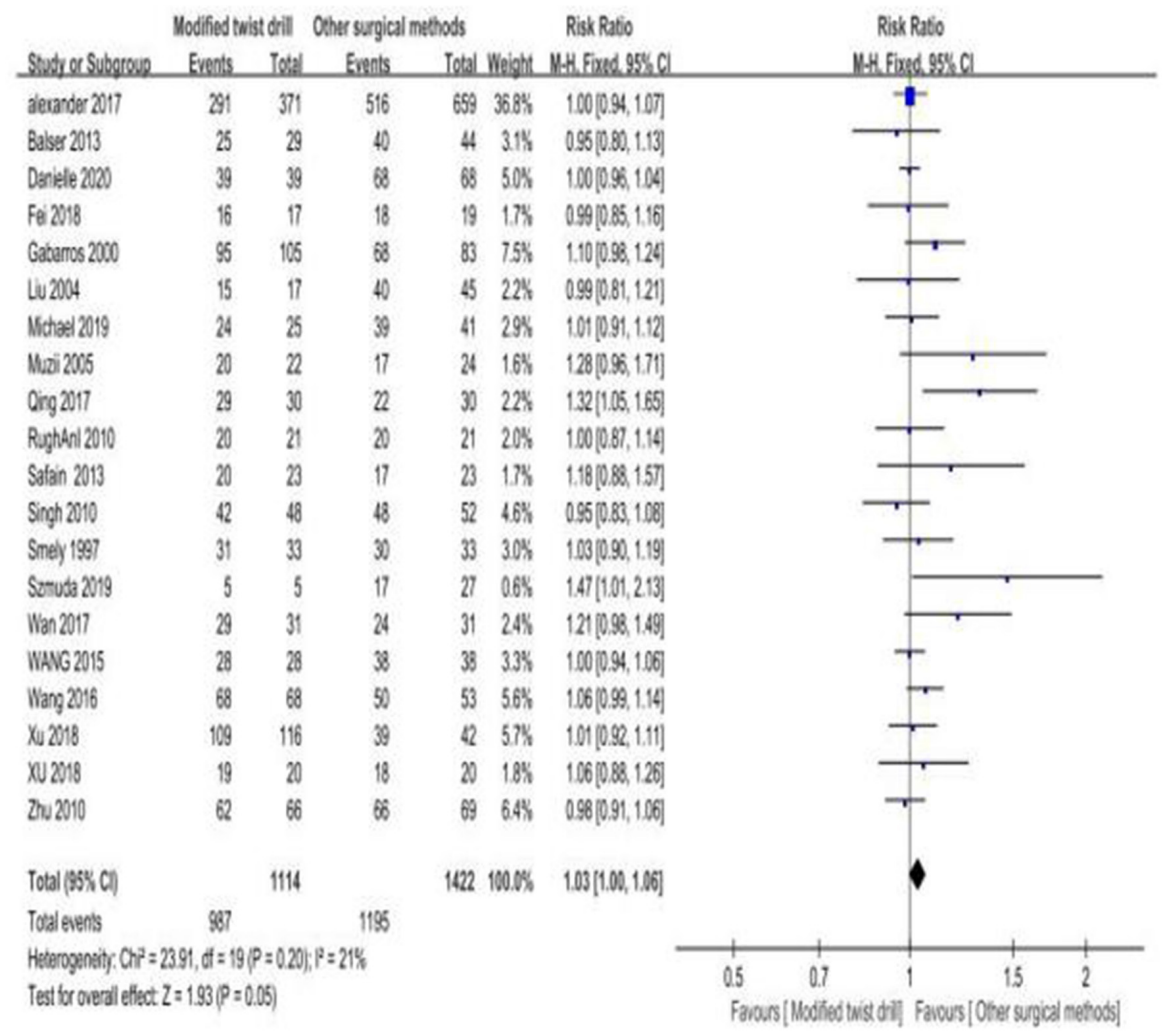
Comparison of surgical success rate between the two groups.

1) Surgical success rate: a total of 20 studies mentioned the surgical success rate, and there was no statistical heterogeneity among the studies (*I*^2^ = 21% < 50%, *P* = 0.20 > 0.1). The fixed-effect model was employed for effect size combination. The results showed that the surgical success rate of TDC with hollow screws was 1.03 times that of BHC, but there was no statistical significance (RR = 1.03, *P* = 0.05; Figure 3). Since the type of puncture needle could affect the combined outcome measure (success rate), subgroup analysis was performed.

**Figure 4:**
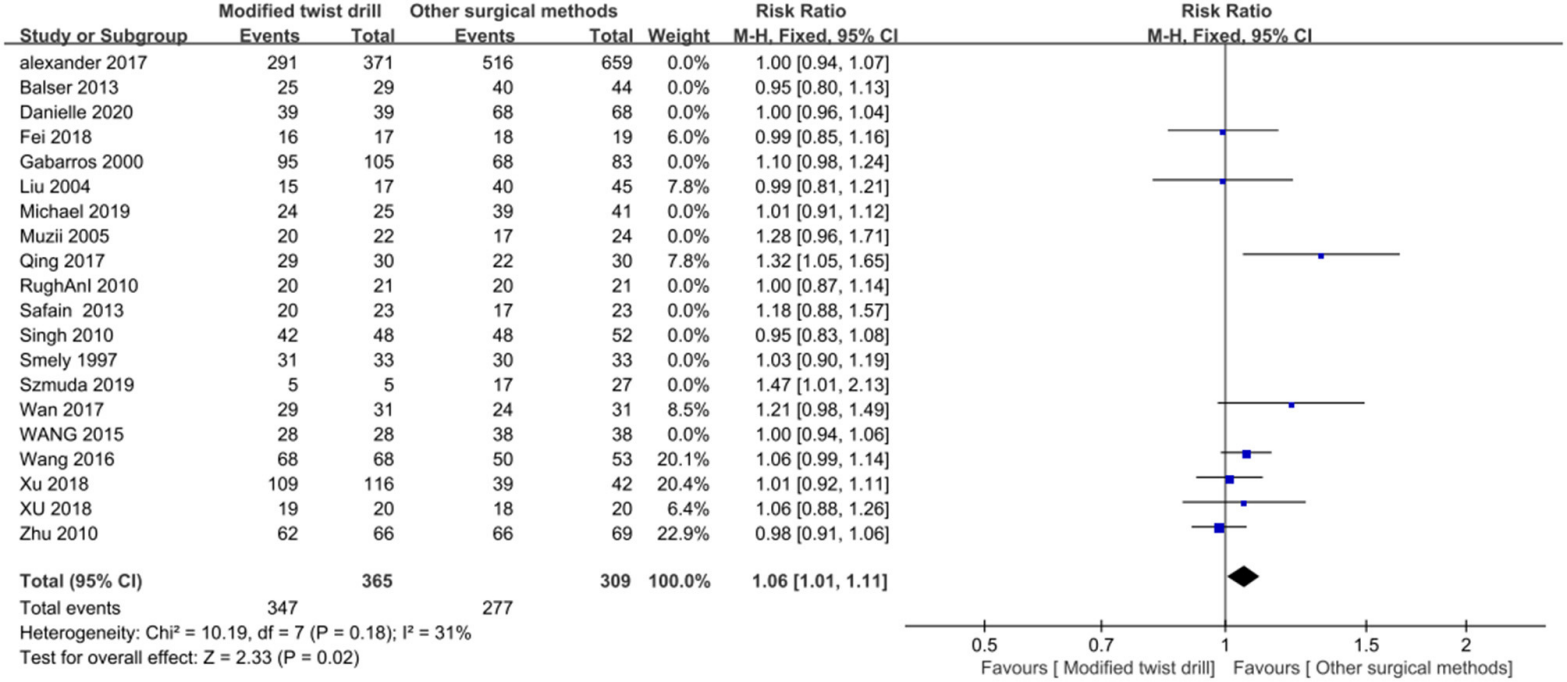
Comparison of surgical success rate between YL-1 group and trepanation group.

Subgroup analysis: The puncture needle used in 8 of 20 included studies was the YL-1 needle, and there was no statistical heterogeneity among the eight studies (*I*^2^ = 31% < 50%, *P* = 0.18). A fixed-effect model was chosen for combination, and the results showed that the surgical success rate of the YL-1 needle was 1.06 times that of BHC, with statistical significance (RR = 1.05, *P* = 0.02; Figure 4).

**Figure 5:**
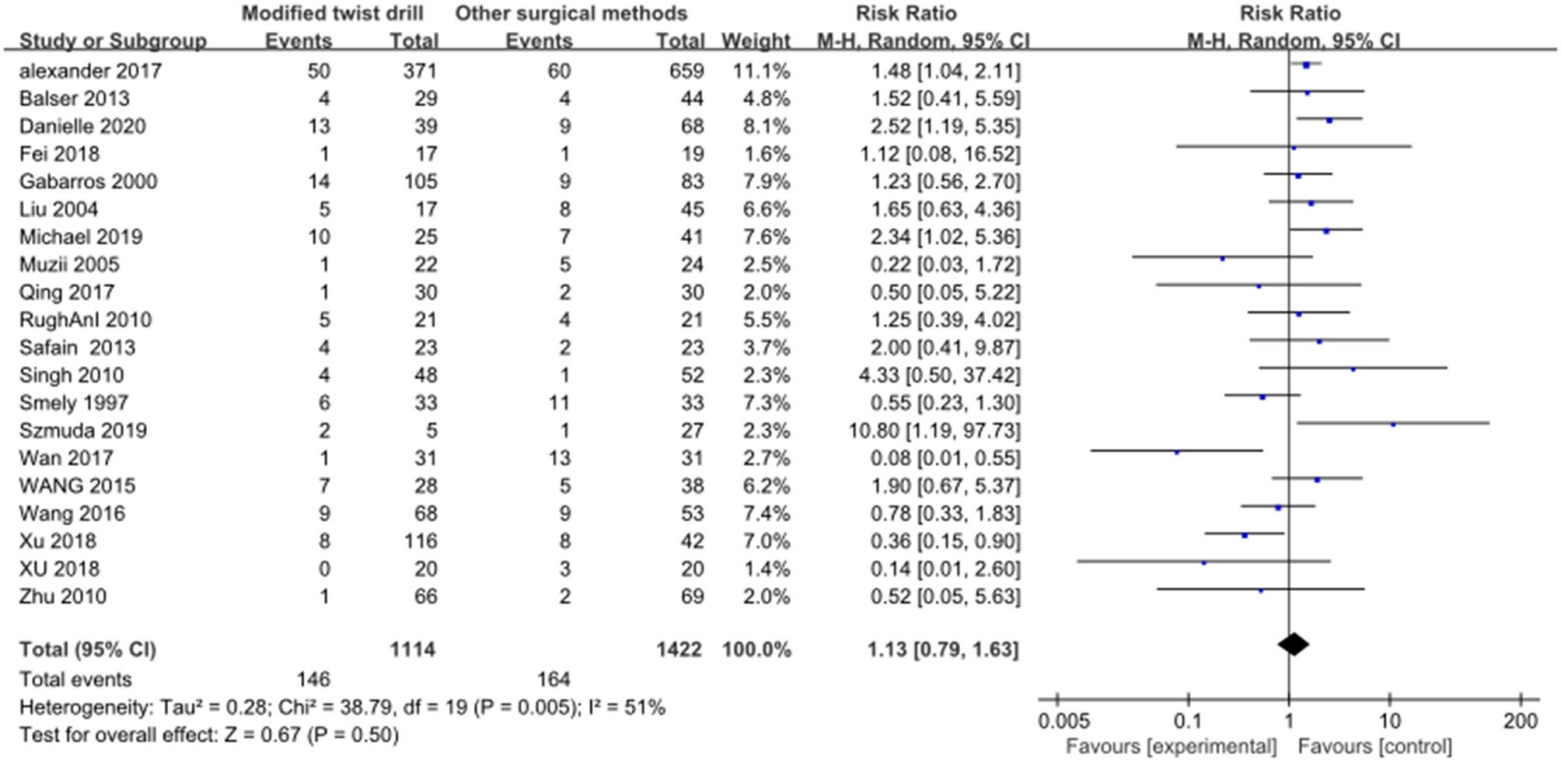
Comparison of postoperative recurrence rate between the two groups.

2) Postoperative recurrence rate: A total of 20 studies reported postoperative recurrence rate, and there was statistical heterogeneity among the studies (*I*^2^ = 51% > 50%, *P* = 0.005 < 0.1). A random-effect model was employed for combination. Results: The recurrence rate of TDC with hollow screws was 1.13 times that of BHC, with no statistical significance (RR = 1.13, *P* = 0.50; Figure 5). There was mild to moderate heterogeneity among the included studies in this review. In addition to using a random-effect combination, it was also necessary to explore the root of heterogeneity.

**Figure 6:**
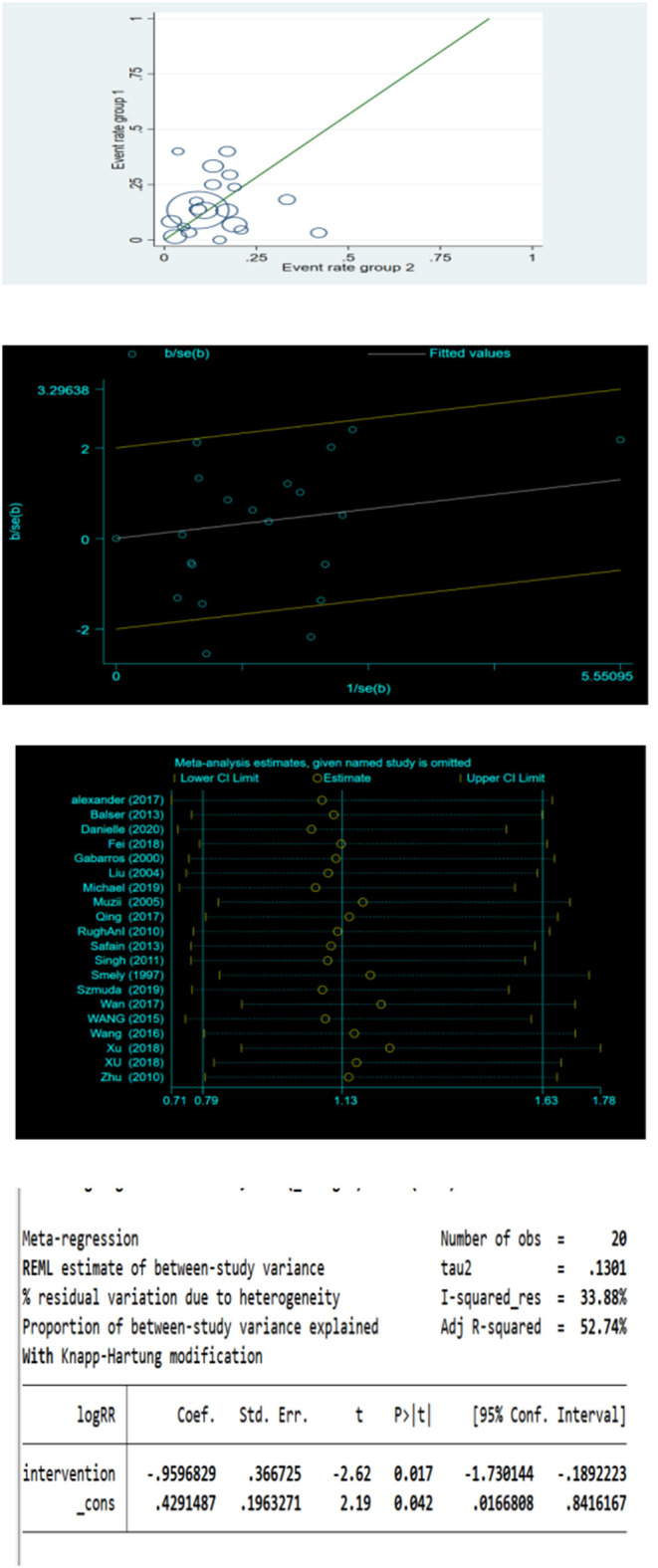
L'Abbe plot, radial plot, sensitivity analysis and meta regression for postoperative recurrence rate.

Sensitivity analysis: The results of the L'Abbe and radial plots suggested that some studies had the possibility for heterogeneity (Figure 6). The sensitivity analysis did not find studies with a significant effect on heterogeneity (Figure 6). Subsequently, meta-regression was conducted, where the patients were divided into the YL-1 needle group and other puncture needle groups based on the intervention measures of the covariates. The results showed that the control measures were the source of heterogeneity (Figure 6). Therefore, subgroup analysis was performed.

Subgroup analysis: There was no statistical heterogeneity among the studies in the YL-1 needle group (*I*^2^ = 41% < 50%, *P* = 0.105 > 0.1) and in other puncture needle groups (*I*^2^ = 32% < 50%, *P* = 0.135). A fixed-effect model was used.

**Figure 7:**
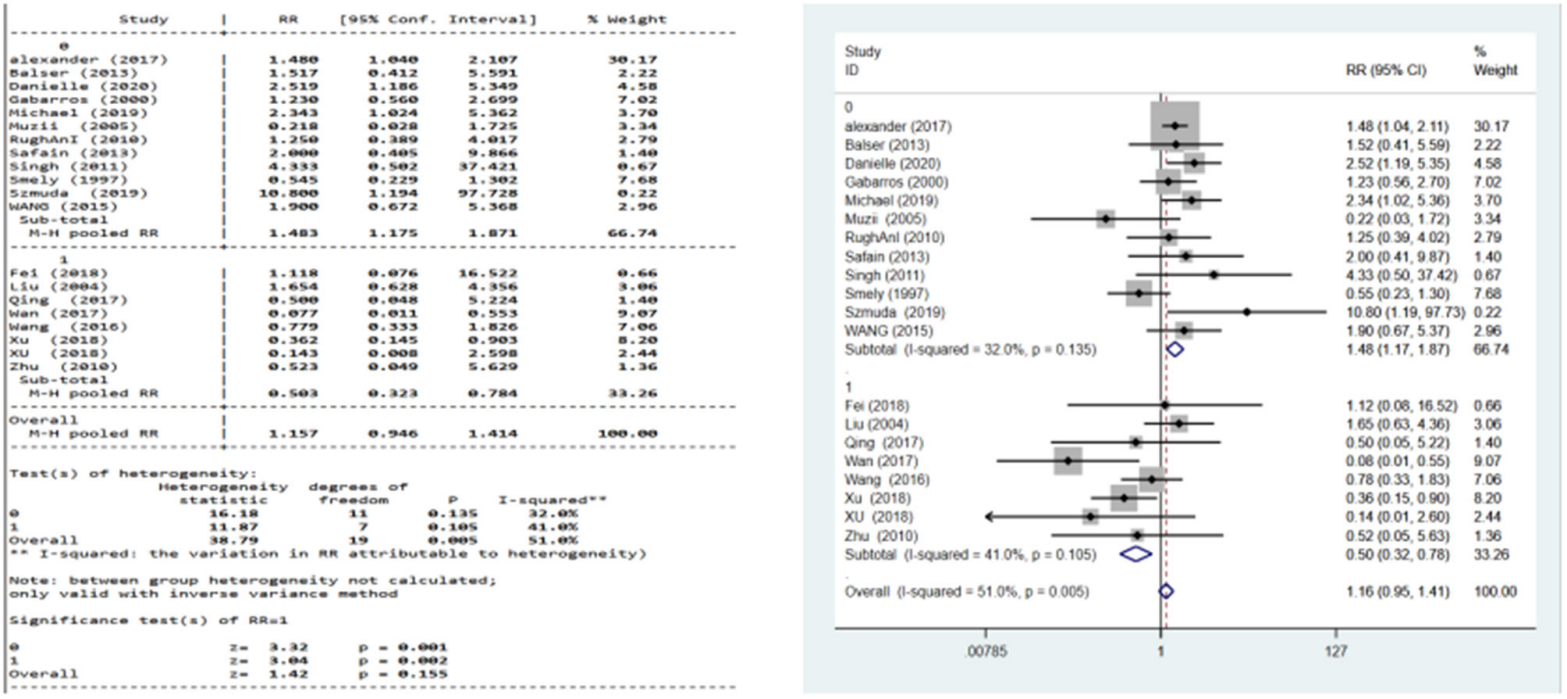
Subgroup analysis for postoperative recurrence rate.

Results: The recurrence rate of the YL-1 needle group was 50.3% that of BHC, with statistical significance (RR = 0.503, P = 0.002; Figure 7). The recurrence rate of other puncture needle groups was 1.483 times that of BHC, with statistical significance (RR = 1.483, *P* = 0.001; Figure 7).

3) Postoperative complications: A total of 19 included studies mentioned postoperative complications, without statistical heterogeneity among the studies (*I*^2^ = 20% < 50%, *P* = 0.21). A fixed-effect model was used. Results of meta-analysis: the postoperative complications of TDC with hollow screws were 57% that of the BHC group, which was much lower, with statistical significance (RR = 0.57, *P* = 0.0002; Supplementary Materials).

4) Acute intracranial hemorrhage: A total of 13 studies contained relevant data, and there was no statistical heterogeneity among the studies (*I*^2^ = 0.0% < 50%, *P* = 0.861). Using the fixed-effect model, the results showed that the acute intracranial hemorrhage rate after TDC with hollow screws was 58.4% that of BHC, which was much lower, with statistical significance (RR = 0.584, *P* = 0.027; Supplementary Materials).

5) Mortality: A total of 18 studies reported mortality, and there was no statistical heterogeneity among the studies (*I*^2^ = 0% < 50%, *P* = 0.68 > 0.1). The fixed-effect model was used. Results: the mortality of TDC with hollow screws was slightly higher than that of BHC, but there was no statistical significance (OR = 1.01, P = 0.95 > 0.05; Supplementary Materials). Heterogeneity and meta-analysis were performed for YL-1 needle groups, and the results were similar (Supplementary Materials).

6) Length of hospital stay: A total of eight studies were included, and there was statistical heterogeneity among the studies (*I*^2^ = 99.2% > 50%, *P* < 0.001). The random-effect model was used. The results showed that the hospital stay for TDC with hollow screws was significantly shorter by 3.752 days, with statistical significance (WMD = −3.752, *P* < 0.001; Supplementary Materials).

Sensitivity analysis and meta-regression: No study with a significant effect on heterogeneity was found in the sensitivity analysis (Supplementary Materials); therefore, the patients were divided into YL-1 needle and other puncture needle groups according to the intervention measure. No obvious source of heterogeneity was found in the meta-regression results (Supplementary Materials). Considering that the type of puncture needle could affect the combined outcome measure (length of hospital stay), subgroup analysis was performed.

Subgroup analysis: The intervention measure with YL-1 needles was used by six studies, and these studies had statistical heterogeneity (*I*^2^ = 96.6% > 50%, *P* = 0.000 < 0.1). The random-effect model was used. Results: the length of hospital stay in the YL-1 needle group was more than 5 days shorter than that of the BHC group, with statistical significance (WMD = −5.032, *P* < 0.001; Supplementary Materials). The other two studies had no statistical heterogeneity (*I*^2^ = 25% < 50%, *P* = 0.248), and the results using fixed-effect model showed that the length of hospital stay in the other puncture needle group was 1.804 days less than that of the BHC group (WMD = −1.804, *P* < 0.001; Supplementary Materials).

7) Bias test: The funnel plot and Egger's test were plotted for the surgical success rate, recurrence rate, complications, acute intracranial hemorrhage, mortality, hospital stay, and subgroup analyses. It was found that only the surgical success rate had publication bias (*P* = 0.019, Supplementary Materials). Trim-and-fill method was used for correction. After four iterations, the results of three studies were simulated. The effect size of 23 studies after combination was 1.019, which was not significantly reversed compared with the results before the trim-and-fill, and the results of the meta-analysis were stable (Supplementary Materials). The remaining studies had no publication bias (Supplementary Materials).

## Discussion

Patients with CSDH having definite clinical symptoms, large hematoma volume, and no surgical contraindications need to undergo surgical treatment. Traditional neurosurgical modalities include craniostomy hematoma evacuation, BHC in the operating room, and TDC. Hematoma evacuation using craniotomy is no longer a primary option in clinical practice due to its high mortality rate (as high as 29%) (33). It is only considered in a few patients with intractable CSDH or severe capsular thickening and calcification. With further study of the pathogenesis of CSDH, McKissock et al. successfully cured patients with CSDH using BHC in the operating room for the first time. Thus, BHC became the first choice for the treatment of CSDH in clinical practice because of its simple operation, high success rate, and relatively better mortality rate (34). However, it also possesses several notable disadvantages in clinical practice, which include general anesthesia, large operating area, poor tolerance in elderly patients, acute intracranial hemorrhage, and high postoperative recurrence rate. Recently, TDC with hollow screws (e.g., SEPS, the hollow screw, and YL-1 needle), which is a bedside procedure, has been favored by frontline physicians. Although many studies compared the efficacy and safety of TDC with hollow screws and BHC in the treatment of CSDH, the results were not consistent (9, 10, 12, 16). Additionally, although there are evidence-based studies on minimally invasive punctures, they are either old or do not consider the different needle types. Hence, this review comprehensively assessed the efficacy and safety of the two groups by combining and comparing the relevant data between the two groups of TDC with hollow screws and BHC.

The surgical success rate is a significant indicator for evaluating the efficacy of surgery. This review indicated that the success rate of TDC with hollow screws was slightly higher than that of the BHC group, but the difference was not statistically significant, which is also in line with most results of previous studies. The results of subgroup analysis suggested that the surgical success rate of the YL-1 needle was slightly higher than that of the BHC group with statistical significance.

The recurrence of hematoma is also a difficult problem in the treatment of CSDH. Reducing the recurrence is equal to reducing the complications and medical costs. The results of this review showed that the recurrence rate of TDC with hollow screws was similar to that of the BHC group. The results of subgroup analysis suggested that the recurrence rate of the YL-1 needle was only about half that of the BHC group. Postoperative complications of CSDH are an important cause of postoperative reduced quality of life. We first analyzed the total complication rate. The results showed that the rate of TDC with hollow screws was much lower than that of BHC, approximately half that of the BHC group. Common postoperative complications of CSDH include intracranial hemorrhage, seizures, and infection. Seizures are the most common complication of CSDH. As with other factors stimulating the cortex, patients could develop seizures due to the presence of a hematoma and possible changes in the amount of hematoma (35). Therefore, many surgeons use antiepileptic drugs in the perioperative period to reduce the risk of seizures due to changes in intracranial pressure, traumatic brain injury, and possible postoperative bleeding based on clinical experience. Multivariate logistic regression analysis by Grobelny et al. (36) also demonstrated that preoperative antiepileptic drugs were the only factor responsible for postoperative seizure reduction. Due to the above clinical experience and related studies, many medical institutions routinely use antiepileptic drugs to prevent seizures after surgery, and many studies lack the record of antiepileptic dose and duration. Since antiepileptic drugs prevent seizures, we did not include seizures as an outcome measure. As frontline neurosurgeons in clinical practice, we were more worried about acute postoperative intracranial hemorrhage, which is an important cause of poor surgical results. The results of this review showed that the rate of acute intracranial hemorrhage was much lower in TDC with hollow screws. Moreover, the mortality of TDC with hollow screws group was also lower than that of the BHC group.

The results of this review indicated that the length of hospital stay was significantly lower for TDC with hollow screws than for BHC. The subgroup analysis showed that the length of hospital stay was significantly shorter in the YL-1 needle group than in the BHC group. Combined with the findings above and surgical experience, TDC with hollow screws has the following advantages: 1) The puncture needle that integrates the drilling can directly reach the hematoma with less bleeding and injury after accurate preoperative positioning, thus reducing the patient's pain; 2) TDC with hollow screws is easy to operate, and it can be implemented at the bedside with local anesthesia, thereby avoiding general anesthesia and shortening the length of hospital stay; 3) The puncture needle is short and penetrates the dural layer, which avoids brain tissue injury. In addition, the puncture needle has good airtight drainage, which reduces the incidence of intracranial infection and pneumocephalus, thus reducing complications; 4) There are no special surgical contraindications, and it is suitable for CSDH patients that are older or in poor conditions; 5) The surgical equipment is simple and cheap; 6) Although the puncture needle has a small aperture and is easily blocked by blood clots resulting in incomplete drainage, the closed drainage system of the YL-1 needle contains a three-way valve that can be injected with urokinase to dissolve the blood clot after surgery. Altogether, we found that the above might contribute to its lower recurrence rate.

The limitations of this review were as follows:

1) Although several cases of the case-control studies met the inclusion criteria, the intervention group was quite small.
2) While extensive searches were carried out in many databases, we eventually only included studies in Chinese and English, which could lead to selection bias. In addition, three Chinese studies were included, which could have led to a regional deviation in the final conclusion.
3) When the hospitalization time was combined, there was great heterogeneity, and the significant heterogeneity could be derived from clinical heterogeneity, including the patient population (e.g., age, education level, and underlying diseases), the size and location of the initial hematoma, different perioperative management, and the operation proficiency of surgeons, and the inconsistent discharge criteria in each medical institution.

## Conclusion

In summary, TDC with hollow screws is a relatively effective first-line treatment for CSDH. TDC with hollow screws has a lower incidence of acute intracranial hemorrhage, overall complication rate, and length of hospital stay. Nevertheless, further confirmation using a larger sample of multicenter, high-quality clinical randomized controlled trial is warranted.

## Data Availability Statement

The original contributions presented in the study are included in the article/Supplementary Material, further inquiries can be directed to the corresponding author.

## Author Contributions

ZW: conception and design and manuscript writing. CW: administrative support. ZW and HJ: provision of study materials or patients. ZW, YW, and HJ: collection and assembly of data. ZW and YW: data analysis and interpretation. All authors final approval of manuscript.

## Funding

This work was supported by Doctor Start-up Fund (No. BSQDJ0130). PROSPERO Registration Number: CRD42021270835.

## Conflict of Interest

The authors declare that the research was conducted in the absence of any commercial or financial relationships that could be construed as a potential conflict of interest.

## Publisher's Note

All claims expressed in this article are solely those of the authors and do not necessarily represent those of their affiliated organizations, or those of the publisher, the editors and the reviewers. Any product that may be evaluated in this article, or claim that may be made by its manufacturer, is not guaranteed or endorsed by the publisher.
