# Supplementary material for: Effect of Twist-Drill Craniostomy With Hollow Screws for Evacuation of Chronic Subdural Hematoma: A Meta-Analysis
Source: Front Neurol. 2022 Jan 28;12:811873. doi: 10.3389/fneur.2021.811873 (PMC8833031; doi:10.3389/fneur.2021.811873)
Supplement: Supplementary file 1 [file Data_Sheet_1.docx]

| 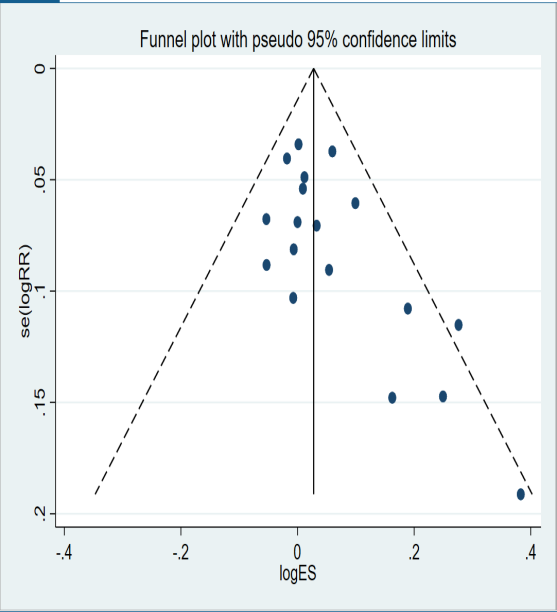 | 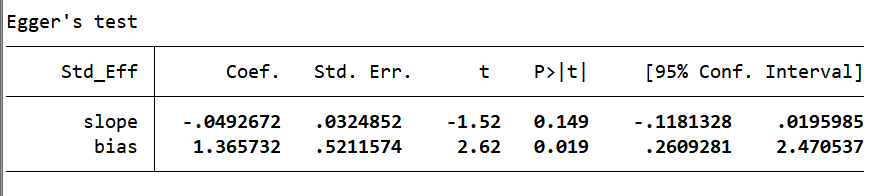 |
| --- | --- |

Figure 16 funnel plot for publication bias of surgical success rate


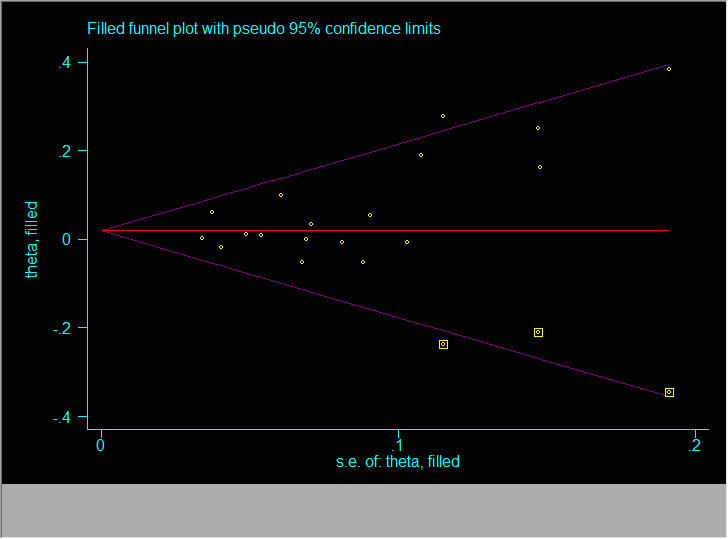


Figure 17 Clipping results of surgical success rate

| 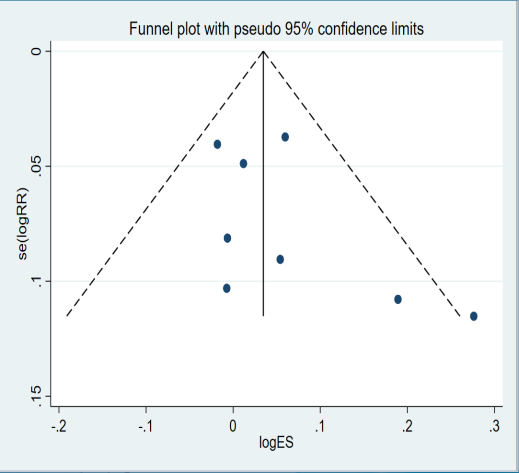 | 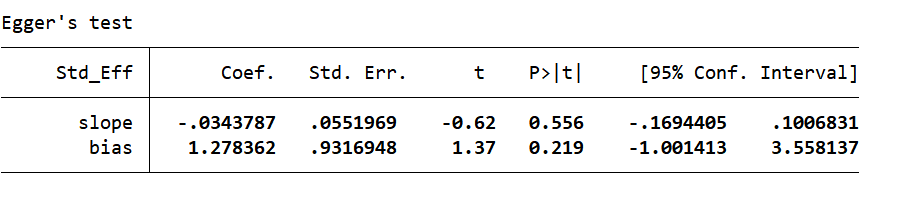 |
| --- | --- |

Figure 18 funnel plot for publication bias of surgical success rate in the YL-1 group

| 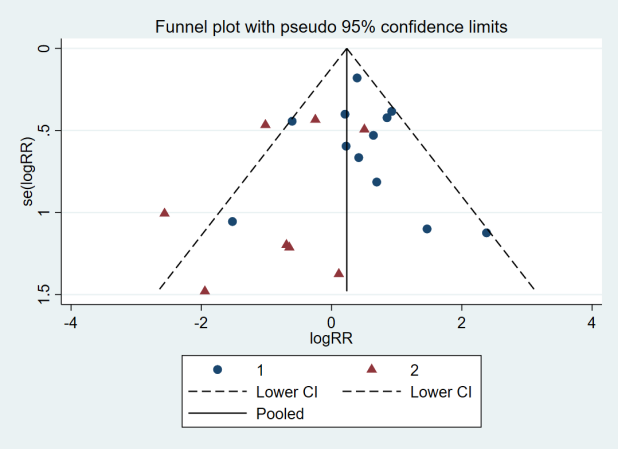 | 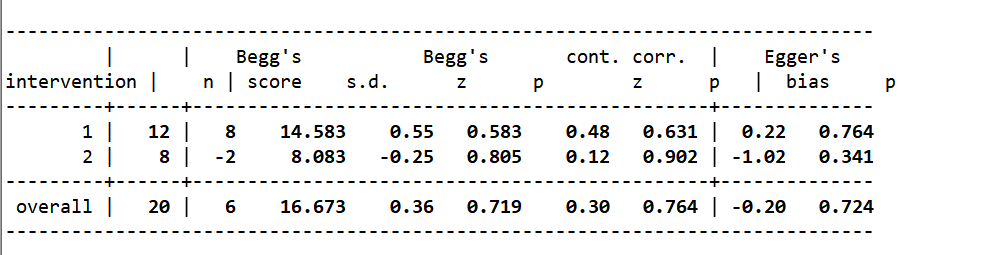 |
| --- | --- |

Figure 19 funnel plot for publication bias of postoperative recurrence rate

| 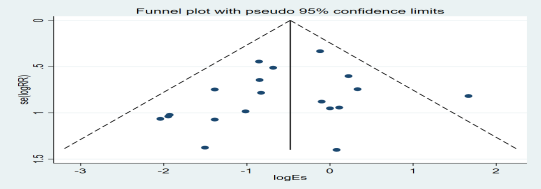 |
| --- |
| 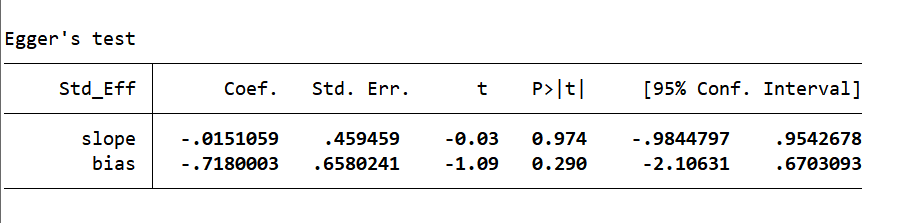 |

Figure 20 funnel plot for publication bias of postoperative complications

| 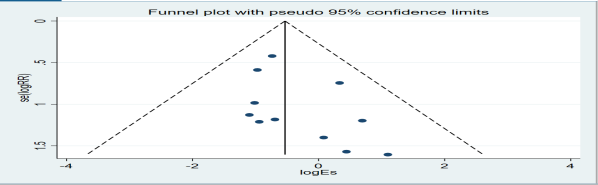 |
| --- |
| 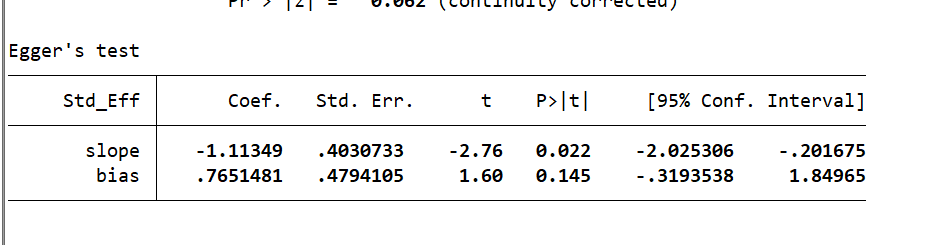 |

Figure 21 funnel plot for publication bias of acute intracranial hemorrhage

| 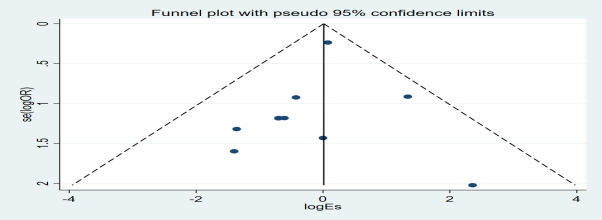 | 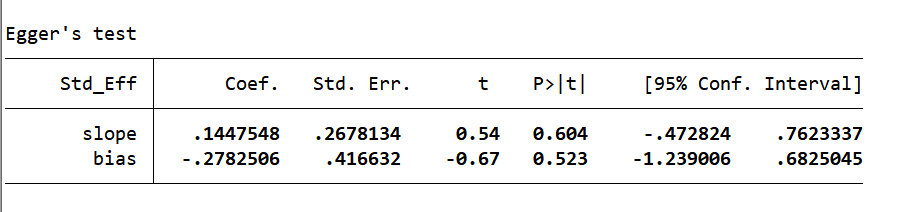 |
| --- | --- |

Figure 22 funnel plot for publication bias of mortality

| 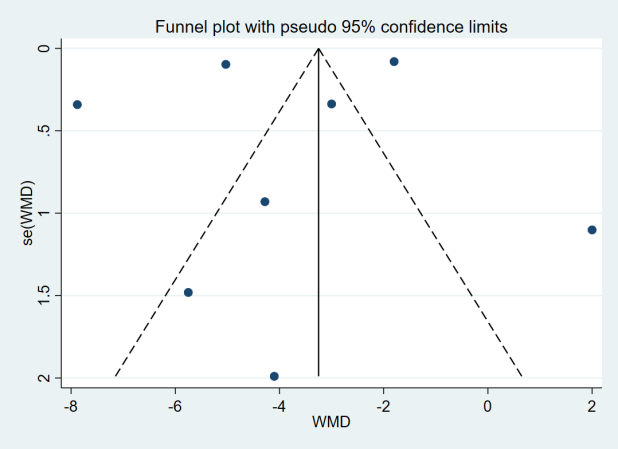 | 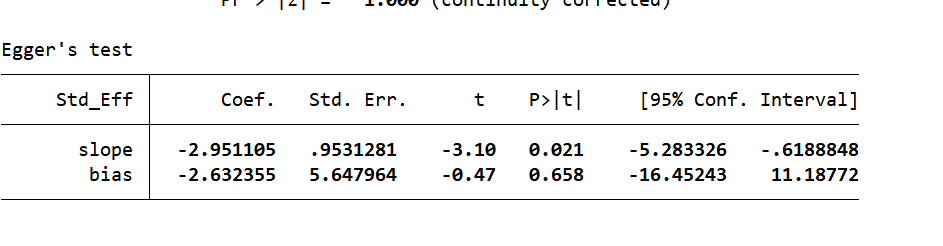 |
| --- | --- |

Figure 23 funnel plot for publication bias of length of hospital stay


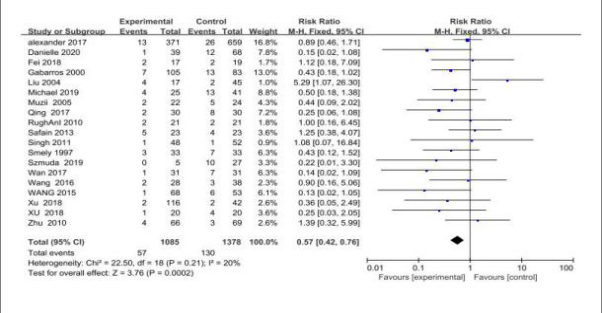


Figure 8 comparison of postoperative complications between the two groups


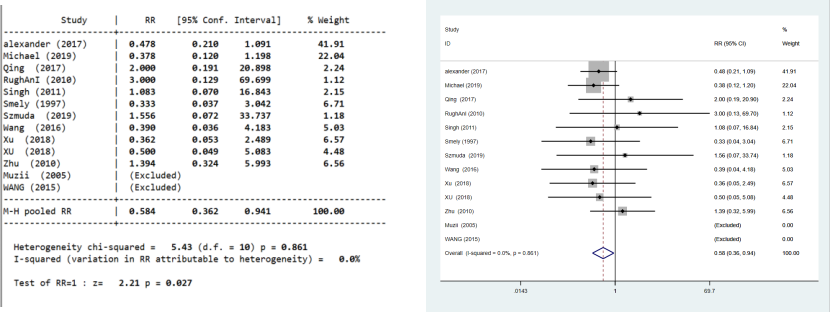


Figure 9 comparison of acute intracranial hemorrhage between the two groups


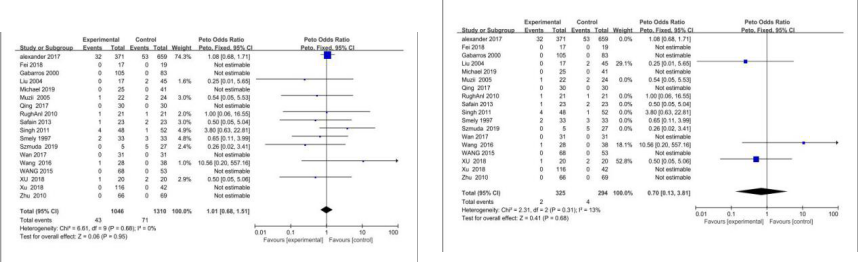


Figure 10 comparison of mortality between the two groups


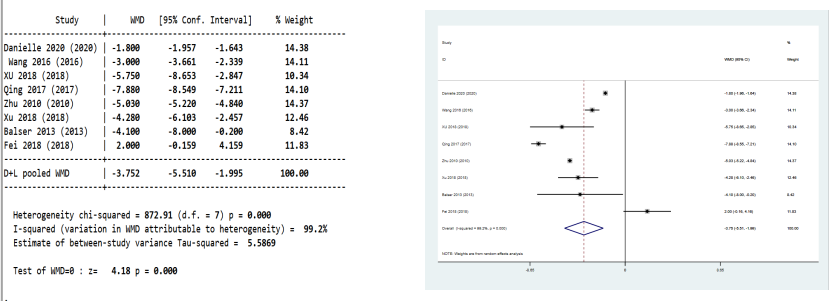


Figure 11 comparison of length of hospital stay between the two groups


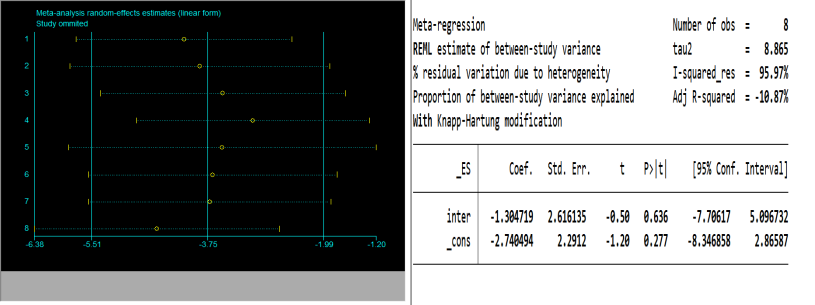


Figure 12 sensitivity analysis and meta regression for length of hospital stay


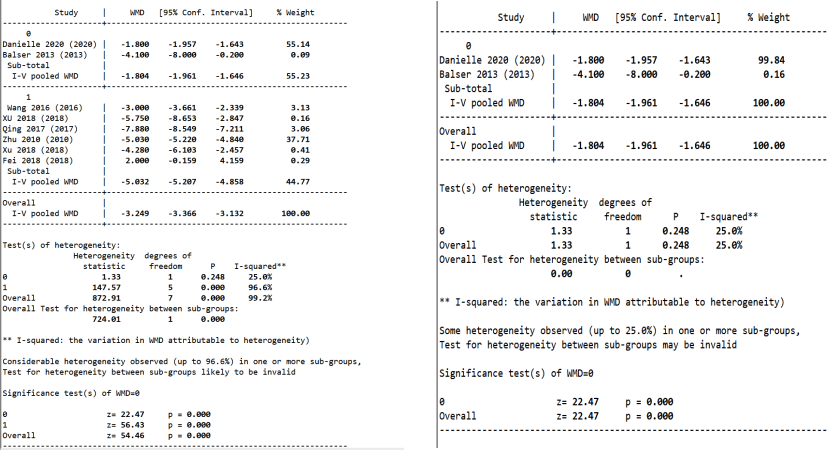


Figure 13 subgroup analysis for length of hospital stay
